# A Three-Step Approach with Adaptive Additive Magnitude Selection for the Sharpening of Images

**DOI:** 10.1155/2014/528696

**Published:** 2014-09-16

**Authors:** Lih-Jen Kau, Tien-Lin Lee

**Affiliations:** Department of Electronic Engineering, National Taipei University of Technology, No. 1, Section 3, Chung-Hsiao E. Road, Taipei 10608, Taiwan

## Abstract

Aimed to find the additive magnitude automatically and adaptively, we propose a three-step and model-based approach for the sharpening of images in this paper. In the first pass, a Grey prediction model is applied to find a global maximal additive magnitude so that the condition of oversharpening in images to be sharpened can be avoided. During the second pass, edge pixels are picked out with our previously proposed edge detection mechanism. In this pass, a low-pass filter is also applied so that isolated pixels will not be regarded as around an edge. In the final pass, those pixels detected as around an edge are adjusted adaptively based on the local statistics, and those nonedge pixels are kept unaltered. Extensive experiments on natural images as well as medical images with subjective and objective evaluations will be given to demonstrate the usefulness of the proposed approach.

## 1. Introduction

The purpose of image sharpening is to highlight transitions or discontinuities in intensity. As it is widely applied in medical image processing, electronic printing, as well as industrial applications such as defect inspections, many research results regarding the image sharpening approaches have been proposed [1–19]. A commonly used technology for the sharpening of images includes first finding the second derivative, for example, using Laplacian filter, of the original image and then adding the differentiated image to the original one to get a sharpened image [1]. In addition to the method mentioned above, the use of an* unsharp mask* in conjunction with a* highboost filter* for the sharpening of images is also widely used in printing and publishing industry [1]. In [2], a content-adaptive algorithm is proposed for the sharpening of images. By extracting the length of lines in the image to be sharpened, the content characteristic as well as the increment or decrement magnitude to be added to the original image can be determined automatically. Besides, regions with artifacts will be sharpened more, while that of natural objects will be less sharpened [2]. In [3], a so-called intuitionistic fuzzy approach is applied for the edge enhancement, and the resulted image can have a very distinct transition on boundaries for different types of medical images [3]. In [4], color image to be sharpened is first converted to* YIQ* or* CIELAB* color space, and then the method of unsharp masking and fuzzy morphological sharpening will be used to adjust the intensity of pixels around boundaries [4].

Since most of the approaches apply the use of differentiation operators to highlight the discontinuities, that is, an edge or a boundary, within an image, the noise suppression can be an annoying problem during the process of edge enhancement. To conquer this problem an unsupervised edge enhancement algorithm based on a combination of wavelet coefficients at different scales is proposed in [5]. The multiscale algorithm can be successfully applied for automatic edge enhancement and detection in speckled synthetic aperture radar (SAR) images [5]. In [6], a technique combining kernel regression and local homogeneity is proposed. The image to be sharpened is first filtered with kernel regression, and then the local homogeneity computation is introduced for further smoothing so that the algorithm is effective about noise reduction and edge enhancement [6]. In [7], a fuzzy logic approach is applied for the sharpening as well as the enhancement of local contrast so that the problem of noise-sensitive in conventional linear unsharp masking technique can be avoided. In [8], an edge enhancement approach is proposed by using image fusion technique. The image to be processed is first convolved with low-pass and high-pass filters, where the filter parameters are determined based on the histogram of the image to be processed. After that, the filtered images are adjusted based on the statistics of the filtered images and then fused together to get an edge enhanced image [8]. A Sobel operator-based approach for the sharpening of edges in a grey scale image is proposed in [9]. The image to be sharpened is first processed by a Gaussian low-pass filter to get a blurred image, and then the Sobel operator is applied to find the edges in the image as well as the gradient of the pixel to be processed. In [9], the gradient around a pixel is quantized to one of a set of four predefined angles. After that, a non maximum suppression and the so-called* Hysteresis thresholding* approach will be applied for nonedge pixel and salt-and-pepper noises removal respectively. Finally, the thin and smooth edges in the image can be obtained after these processes. In [10], an approach of our previous work which is based on the concept of “Just Noticeable Distortion (JND)” is proposed for the sharpening of color images. The image to be sharpened is first transformed to the HSV color model, and then the edge pixels in the channel of Value will be picked out and used for the process of sharpening. During the process of sharpening, the channel of Hue and Saturation are kept unaltered [10]. Besides, a low-pass filter is applied to the edge pixels in the channel of Value before the sharpening process so that salt-and-pepper noises can be suppressed. Finally, the adjusted channel of Value is integrated with that of Hue and Saturation to get the sharpened color image in [10].

In certain cases, the sharpening of an image is carried out in conjunction with image contrast enhancement [11–19]. In such kind of approaches, the statistics of the image to be sharpened is first obtained, and then the equalization of histogram will be performed. In [11], the Laplace filter is first applied so that the strength of the discontinuity in the image to be processed can be evaluated. After that, a Laplace filter is used again to highlight discontinuity with smaller strength while a Gaussian filter is applied to suppress discontinuity with larger strength. Finally, the contrast will be enhanced by using an adaptive histogram equalization approach to get a better visual perceptual quality [11]. In [12], an edge-weighted contrast enhancement algorithm is proposed. The image to be enhanced is first convolved with a median filter to get a low-pass filtered image. Meanwhile, the original image is also processed by a weighted threshold histogram equalization (WTHE) approach to get an rudimentary enhanced image. Finally, the Sobel operator is applied to the original image to be enhanced to get a couple of weights for the low-passed filtered image as well as the rudimentary enhanced image so that the two images can be merged together to get the final enhanced image.

Though histogram equalization (HE) has been proved to be simple and effective in contrast enhancement, however, it tends to change the mean brightness of the image to the middle level of the permitted range and hence is not very suitable for consumer electronic products, where preserving the original brightness is essential to avoid unnatural look and visual artifacts [13]. To conquer this problem, a brightness preserving histogram equalization with maximum entropy (BPHEME) approach is proposed in [13]. The BPHEME tries to find the target histogram that maximizes the entropy under the constraints that the mean brightness is fixed so that not only the image can be enhanced, but also the original brightness can be preserved as well [13].

Based on the analysis of many of the researches for contrast enhancement, the use of an intensity distribution of the whole image is the major cause of visual artifacts in conventional histogram equalization. Therefore, some of the researches propose the use of a so-called subregion or subimage histogram equalization [14–19]. Instead of using the conventional histogram equalization, a method called subregions histogram equalization is proposed for contrast enhancement in [14]. The image to be enhanced is first convolved with a Gaussian filter to get smoothed intensity values, and then the transformation function is applied for histogram equalization [14]. With the process of convolving with the Gaussian filter, the transformation function used is not based on the intensity of the pixels only, but the values of the neighboring pixels are also considered [14]. In [15], a recursive subimage histogram equalization is developed by iteratively dividing the histogram based on median rather than mean values so that the brightness can be preserved to extend better than previous histogram partitioning methods. A contrast enhancement method using dynamic range separate histogram equalization (DRSHE) is proposed in [16]. The DRSHE first separates the dynamic range of histograms into several parts and resizes the grey scale range of each part based on individual area ratio, and then intensities of histogram in each part are uniformly redistributed in resized grey scale range so that unintended changes in brightness can be suppressed [16]. In [17], an edge-preserving contrast enhancement as well as a multihistogram equalization method is proposed. By utilizing the human visual system, the image to be enhanced is decomposed into segments, resulting in an efficient correction of nonuniform illumination. Additionally, a quantitative measure of image enhancement is also proposed [17]. In [18], an adaptive image equalization algorithm is proposed. The histogram distribution is first synthesized by Gaussian mixture model, and the intersection points of the Gaussian components are used to partition the dynamic range of the image into subintervals. The contrast equalized image is generated by transforming the gray levels in each subinterval according to the dominant Gaussian component and the cumulative distribution function of the subinterval with a weight proportional to the variance of the corresponding Gaussian component. The algorithm is free of parameter setting for a given dynamic range of the enhanced image [18]. Recently, a fuzzy logic-based histogram equalization (FHE) is proposed for intensity transformation and spatial filtering in [19]. The fuzzy histogram is first computed based on fuzzy set theory, and then the fuzzy histogram is divided into two subhistograms based on the median value of the original image. Finally, the two subhistograms are equalized independently to get a brightness preserved and contrast enhanced image. In these histogram-equalized approaches, all the pixels are adjusted, and the intensity as well as the characteristics of the original image changes [11–19].

Instead of using the concept of JND in our previous work [10], we try to determine the maximal additive magnitude Δ with a quite different approach in this paper. To do this, we propose a model-based and three-pass algorithm for the sharpening of grey-scale images. The first pass of the proposed approach is to determine an appropriate maximal additive intensity magnitude Δ based on the global statistics of the image to be sharpened. To highlight the transitions or discontinuities in intensity, pixels around edges or boundaries should be adjusted; that is, an additive magnitude should be imposed on those edge pixels. In general, a larger additive in magnitude can have a better sharpening result; however a larger additive in magnitude can also lead to the saturation of intensity around edge pixels. Aiming to find the maximal additive magnitude automatically for the images to be sharpened, we proposed in this paper the use of a Grey prediction model GM(1,1) so that the condition of over-sharpening or intensity saturation can be avoided. The Grey prediction model is applied in a variety of fields for its ability of generating predicted value of a sequence under limited information or sampled values [20, 21]. With this characteristic, some of the researches apply the Grey prediction model for the edge detection of an image so that the discontinuity or change in intensity can be highlighted [22–24]. Instead of using the Grey prediction model for finding discontinuities in an image directly, in this paper we apply the use of a GM(1,1) model for the determination of the maximal additive value Δ. In this paper, the smallest intensity (*Min*⁡), middle intensity (Mid), and largest intensity (*Max*⁡), as well as the average intensity (Avg) of the image to be sharpened are used as the four sample sequence values of the Grey prediction model so that a predicted value that describes the trend of intensity distribution can be obtained. The maximal additive value Δ is then calculated by subtracting the average intensity value from the Grey predicted value.

During the second pass, pixels around edges or boundaries are picked out with an edge detection mechanism, for example, the well known Canny [25] and Sobel edge detector [1], can be used for this purpose. In this paper, the Canny operator as well as our previously proposed horizontal and vertical differentiator (called HVD for short), which performs difference operation between consecutive pixels in [10], will be used for the detection of an edge. In the third pass, the intensity of those pixels detected as around an edge or boundary are adjusted with an increment or decrement based on our previously proposed local-adapted strategy [10] for the purpose of image sharpening, and that of those nonedge pixels are kept unaltered. With the proposed approach, most of the original information contained in the image can be retained. Actually, the increment or decrement magnitude to be added to an edge pixel is a portion of the largest additive magnitude Δ depending upon the local statistics of the pixel to be adjusted. For this, we first calculate the average intensity of a small local area around the pixel and then check to see if the intensity of the edge pixel is greater than the average intensity value. If so, an increment is added, otherwise a decrement should be added. Finally, a scaling factor can also be used for the adjustment of the additive magnitude in the proposed approach. As we will see in the experiments, the proposed approach can have a very distinct intensity transition for pixels around edges in the sharpened images, which demonstrates the usefulness of the proposed approach.

The rest of the paper is organized as follows.  Section 2 gives an overview of the Grey prediction algorithm. The proposed algorithm is introduced in Section 3. Extensive experiments on the proposed method are given in Section 4. A conclusion is given in Section 5.

## 2. The Grey Prediction

Due to its successful applications in a variety of fields, for example, image processing, statistics, medical, military, and business management, the Grey theory, especially the Grey prediction method, has attained more and more attention recently [20, 21]. Unlike most of the conventional prediction mechanisms, the data samples that are to be used as the inputs of a prediction do not have to be equally spaced or sampled in Grey prediction model. Moreover, a very good prediction result can be obtained under limited data samples or information with the use of a Grey prediction mechanism [20, 21]. For this reason, the commonly used GM(1,1) model, that is, a first-order differential equation with single variable, is applied in this paper to predict the trend of intensity distribution in the image to be sharpened, and then the predicted value is used for the determination of a maximal additive value Δ.

By performing a so-called* accumulated generating* process, an irregular data sequence can be made regular with the use of a GM(1,1) prediction model. The accumulated data sequence with regular property is usually referred to as the* Grey generated sequence*. After this process, the* Grey generated sequence* can be used for the modeling or prediction of future data samples. To utilize the GM(1,1) Grey prediction mechanism based on previously sampled data, we define the observation, that is, the previously sampled data sequence, *x* ^(0)^ as

(1) $$\begin{array}{c} x^{\left(0\right)}=\left\{x^{\left(0\right)}\left(1\right),x^{\left(0\right)}\left(2\right),x^{\left(0\right)}\left(3\right),x^{\left(0\right)}\left(4\right),\ldots \right\}. \end{array}$$

With the sampled data sequence *x* ^(0)^, the Grey prediction modeling can be carried out through the following steps [20, 21].

**Step 1 (perform the accumulated generating operation).**

The first step is to perform the so-called* accumulated generating operation (AGO)* of the original sequence to get the* accumulated generating sequence x* ^(1)^ as below:

(2) $$\begin{array}{c} x^{\left(1\right)}\left(k\right)=\overset{k}{\underset{i=1}{\sum }}x^{\left(0\right)}\left(i\right),\;\;k=1,2,\ldots ,n. \end{array}$$

**Step 2 (calculation of the mean sequence).**

The second step is to calculate the mean sequence *z* ^(1)^(*k*) from *x* ^(1)^(*k*)

(3) $$\begin{array}{c} z^{\left(1\right)}\left(k\right)=\frac{x^{\left(1\right)}\left(k\right)+x^{\left(1\right)}\left(k+1\right)}{2},\;\;k=1,2,\ldots ,n-1. \end{array}$$

**Step 3 (calculation of the semiparameters).**

This step, four intermediate parameters *C*, *D*, *E*, and *F* are calculated as below [20, 21]:

(4) $$\begin{array}{c} C=\overset{n}{\underset{k=2}{\sum }}z^{\left(1\right)}\left(k\right),\\ D=\overset{n}{\underset{k=2}{\sum }}x^{\left(0\right)}\left(k\right),\\ E=\overset{n}{\underset{k=2}{\sum }}z^{\left(1\right)}\left(k\right)\times x^{\left(0\right)}\left(k\right),\\ F=\overset{n}{\underset{k=2}{\sum }}z^{\left(1\right)}{\left(k\right)}^{2}. \end{array}$$

**Step 4 (calculation of the coefficients *a*, *b*).**

In step four, the so-called* developing coefficient a* and* Grey input b* are calculated as below [20, 21]:

(5) $$\begin{array}{c} a=\frac{CD-\left(n-1\right)E}{\left(n-1\right)F-C^{2}},\\ b=\frac{DF-CE}{\left(n-1\right)F-C^{2}}. \end{array}$$

**Step 5 (construct the White response equation).**

With the above parameters, the GM(1,1) prediction can then be modeled by the following:

(6) $$\begin{array}{c} x^{\left(0\right)}\left(k\right)+az^{\left(1\right)}\left(k\right)=b. \end{array}$$

Differentiating with the time index, we have the* Grey differential equation* below

(7) $$\begin{array}{c} \frac{dx^{\left(1\right)}}{dt}+ax^{\left(1\right)}=b. \end{array}$$

The solution of the above equation is called the* White response*, and is given by

(8) $$\begin{array}{c} x^{\left(1\right)}\left(k+1\right)=\left[x^{\left(0\right)}\left(1\right)-\frac{b}{a}\right]\times e^{-(ak)}+\frac{b}{a}. \end{array}$$

**Step 6 (perform inverse accumulated generating operation).**

After the (*k* + 1)th sample of the accumulated generating sequence, that is, *x* ^(1)^(*k* + 1), has been calculated in (8), the (*k* + 1)th sample of the original data sequence can be obtained by using a back-stepping or inverse accumulated generating operation (IAGO) as below

(9) $$\begin{array}{c} x^{\left(0\right)}\left(k+1\right)=x^{\left(1\right)}\left(k+1\right)-x^{\left(1\right)}\left(k\right). \end{array}$$

## 3. Proposed Image Sharpening Algorithm

In this section, the proposed three-pass image sharpening algorithm is to be introduced. The determination of the maximal additive magnitude Δ will be first introduced, and then the edge detection mechanism in [10] will be given after that. Finally, the edge sharpening algorithm will be explained [10].

### 3.1. Maximal Additive Determination-Step I

**Figure 1:**
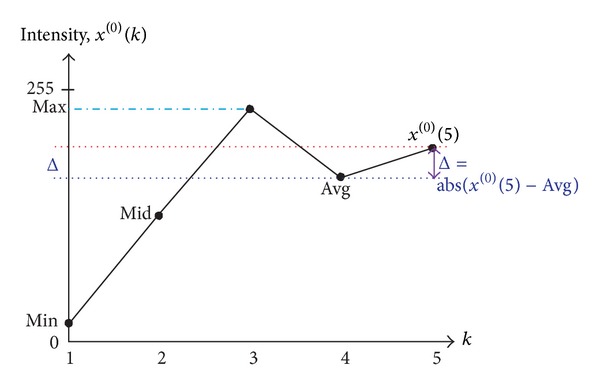
The calculation of Δ based on GM(1,1) prediction model.

The first pass of the proposed approach is to determine an appropriate maximal additive intensity magnitude Δ based on the global statistics of the image to be sharpened. For this, a commonly used Grey prediction model GM(1,1) will be applied for this purpose [20, 21]. To find the Δ, we use the smallest intensity (*Min*⁡), middle intensity (Mid), and largest intensity (*Max*⁡), as well as the average intensity value (Avg) of the image to be sharpened as the four sample sequence values, that is, *x* ^(0)^(1),…, *x* ^(0)^(4), of the Grey prediction model [20, 21]. The four sample data values of the original sequence *x* ^(0)^ are then used to predict the fifth sample *x* ^(0)^(5) in the sequence, which describes the trend of intensity distribution of the image to be sharpened (as illustrated in Figure 1). The maximal additive value Δ is then determined by

(10) $$\begin{array}{c} \Delta =\left|x^{\left(0\right)}\left(5\right)-\text{Avg}\right|. \end{array}$$

Recall that the purpose of image sharpening is to highlight the discontinuities. For this, an increment or decrement should be added with the original intensity for pixels around boundaries. A larger additive value usually can have a better sharpening result. However it can also lead to the intensity saturation or the effect of over-sharpening of edge pixels. Therefore, the determination of an appropriate or maximal additive value has become an important step during the sharpening procedure. The Δ given above turns out to be a very good choice in which the intensity saturation phenomenon can be avoided as we will see in the experiment.

### 3.2. Edge Detection and Low Pass Filtering-Step II

In the second pass, pixels around edges are to be picked out. The commonly used* Canny* and* Sobel operator* can be applied for this purpose. Nevertheless, in this paper we apply a very simple algorithm of our previous work in [10] for discontinuity detection by calculating the horizontal and vertical intensity difference of the pixel to be sharpened, that is, the horizontal and vertical differentiator (HVD) [10].

**Figure 2:**
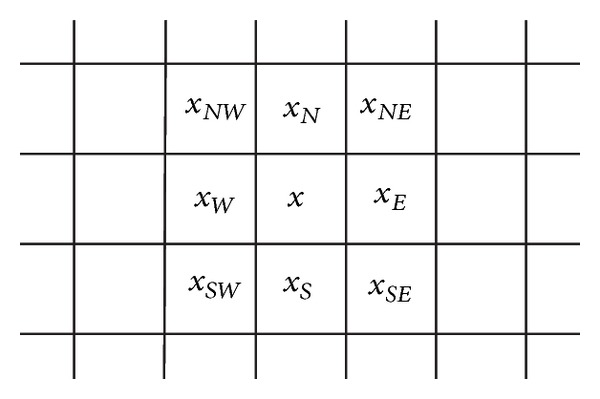
Texture context of the pixel *x* to be sharpened.

By using the HVD, the discontinuity around a pixel *x* can be easily detected by examining the intensity difference between (*x*, *x*_*W*_) and (*x*, *x*_*N*_) (in Figure 2). We then determine if the pixel *x* is around an edge by checking if the first condition of the following equation holds [10]:

(11) $$\begin{array}{c} g\left(x\right)=\{\begin{array}{cc} 1 & \text{if}\;\left|x-x_{W}\right|\geq \theta_{\text{eth}}\;\text{or}\;\left|x-x_{N}\right|\geq \theta_{\text{eth}}\\ 0 & \text{ow}, \end{array} \end{array}$$

where *g* is a bi-level output image with 1*s* represented for edge pixels, and *θ*_eth_ is a predefined threshold which controls the degree of discontinuity that a pixel may be regarded as around an edge [10]. Empirically, a value between 8 to 18 would be a suitable choice for *θ*_eth_.

In this pass, however, some of the isolated pixels can also be detected as around an edge with the proposed HVD edge detector [10]. Therefore, not only the edge information, but also salt-and-pepper noise can be incurred in the bilevel image *g* as well. To avoid this problem, a low-pass filter is applied so that those isolated pixels can be excluded from being regarded as around an edge [10]. To determine if a pixel *x* is really an edge pixel or not, we apply the low-pass filter in [10] and check if the following inequality holds

(12) $$\begin{array}{c} \underset{\forall p\in N_{8}\left(x\right)}{\sum }g\left(p\right)\geq \theta_{\text{Lpf}}, \end{array}$$

where *N*_8_(*x*) means the eight-connected neighbors of pixel *x*. That is, we check the number of 1*s* of the eight neighbors of *x* in the bi-level image *g*. If it is smaller than a predefined threshold *θ*_Lpf_, *x* is regarded as an isolated point, and will be discarded from the list of edge pixels [10]. In this paper, *θ*_Lpf_ is set to be 3. This is due to the fact that the detected edge width would be two-pixel wide if the HVD approach is used, and thus a value of 3 is much suitable for *θ*_Lpf_.

### 3.3. Edge Sharpening Process-Step III

During the final pass, the intensity of those pixels detected as around an edge are adjusted with an increment or decrement based on our previously proposed local-adapted strategy [10] for the purpose of image sharpening, and that of those nonedge pixels are kept unaltered. To highlight smaller discontinuity but keep the image not to be over-sharpened, the additive magnitude has to be adapted to the local statistics of the image. Thus, we first compute the average intensity *LocalMean* of a small local area including the edge pixel *x* to be adjusted and the eight-connected neighbors of *x*, that is, *N*_8_(*x*). We then compare if the intensity of *x* is greater or smaller than the value of *LocalMean*. If the intensity value of *x* is greater than *LocalMean*, an increment *δ*_*x*_ will be added to *x*; otherwise the *δ*_*x*_ will be subtracted from *x* [10]. The additive value *δ*_*x*_ is determined adaptively by

(13) $$\begin{array}{c} \delta_{x}=\{\begin{array}{cc} s\times \Delta \times \left(\frac{x}{LocalMean}\right), & \text{if}\;x<LocalMean;\\ s\times \Delta \times \left(\frac{LocalMean}{x}\right), & \text{ow,} \end{array} \end{array}$$

where *s* is a scaling factor between 0 and 1 that controls the degree of sharpness, and Δ is obtained in (10) [10]. Actually, the term inside the bracket of (13) is for local adaptation which makes the additive magnitude larger for a smaller discontinuity, and vice versa [10]. The sharpened intensity value $\hat{x}$ of *x* is then given by [10]

(14) $$\begin{array}{c} \hat{x}=\{\begin{array}{cc} x-\delta_{x}, & \text{if}\;x<LocalMean;\\ x+\delta_{x}, & \text{otherwise.} \end{array} \end{array}$$

**Figure 3:**
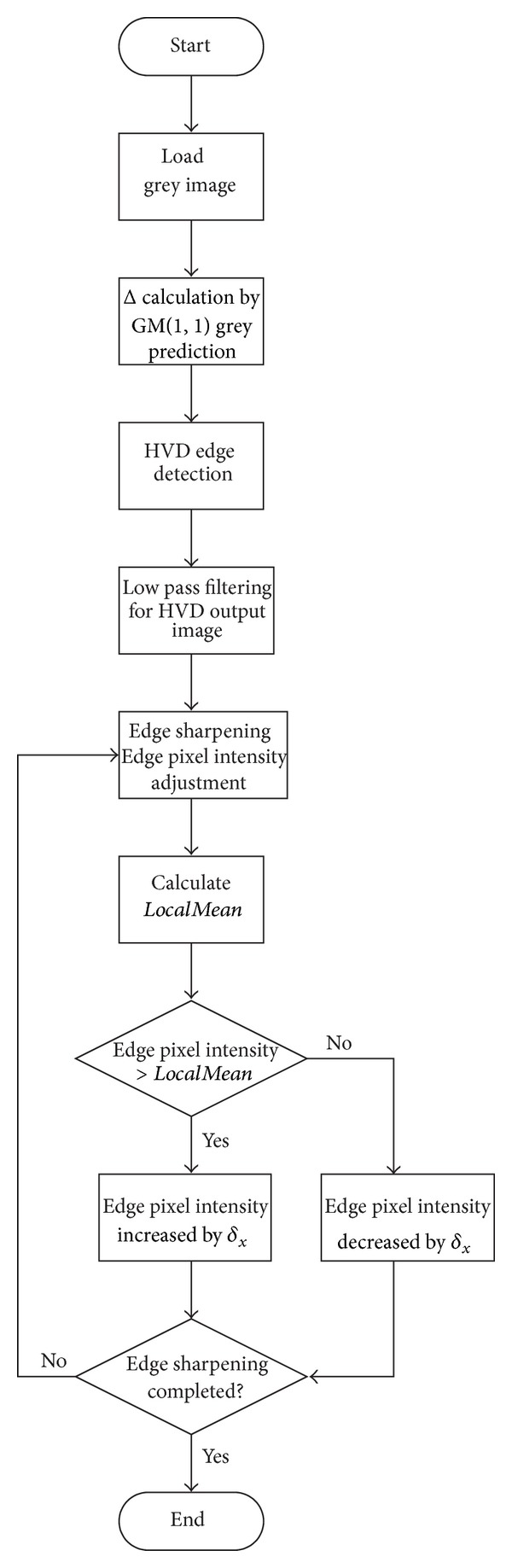
Procedure of the proposed image sharpening algorithm.

As we are dealing with 8-bit grey scale images, the intensity value of each pixel should fall in the range between 0 and 255. Therefore, the sharpened intensity value $\hat{x}$ has to be checked if its value is outside the upper and lower bound. If so, the $\hat{x}$ should be set to 0 or 255 depending on the value smaller than 0 or greater than 255. To summarize, the detailed procedure of the proposed grey-scale image sharpening algorithm is shown in Figure 3.

## 4. Experiments

**Figure 4:**
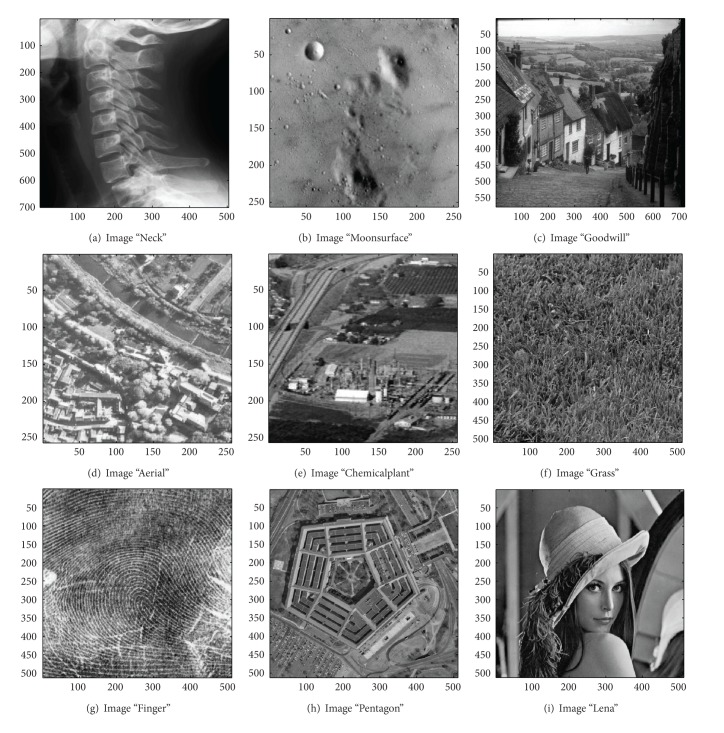
Test images in experiment.

In this section, we evaluate the performance of the proposed image sharpening algorithm. The nine images in Figures 4(a)–4(i) will be used as the test images. Among all the test images, three of which, that is, the image “Aerial,” “Chemicalplant,” and “Pentagon,” are remotely sensed synthetic aperture radar (SAR) images, while the image “Neck” is a medical image, and the others are natural images. Recall that the proposed algorithm is composed of three phases. During the second pass, the* Canny operator* as well as the HVD edge detection approach [10], that is, the horizontal and vertical differentiator, will be used to examine if a pixel is around edges or boundaries.

First of all, a subjective evaluation on the sharpening result will be performed, and then an objective evaluation on the quality will be given. During the objective evaluation process, we compare the sharpened result with the original image and check to see if most of the image content can be preserved after the edge sharpening process. Finally, a complexity analysis on the proposed approach will be given by using the operation count to highlight the efficiency of the proposed algorithm.

### 4.1. Subjective Performance Evaluation

**Figure 5:**
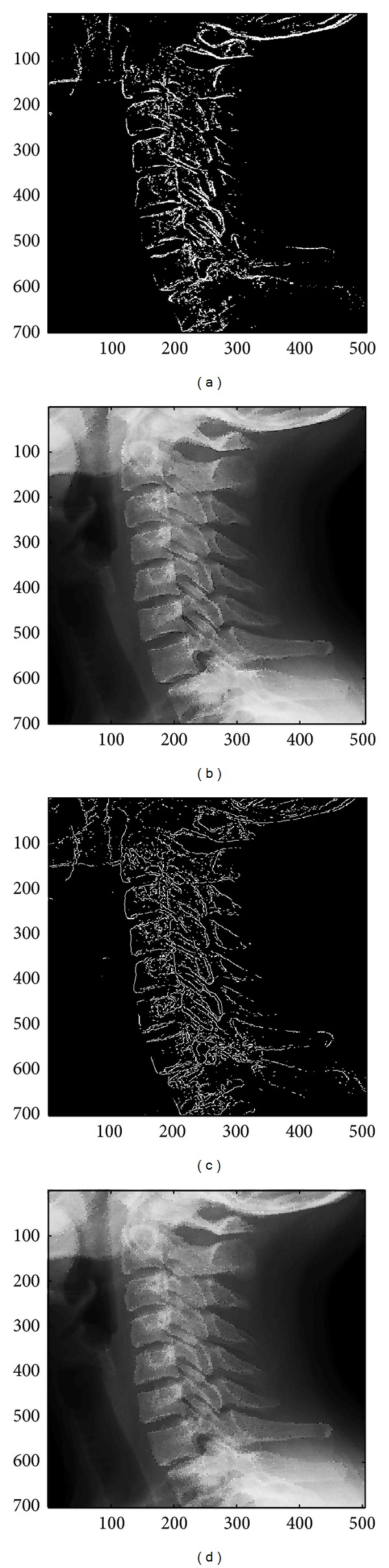
(a) Edges detected by HVD with *θ*_eth_ = 8 in the image “Neck.” (b) Result by HVD with scaling factor *s* = 1.0. (c) Edges detected by* Canny operator *in the image “Neck.” (d) Result by* Canny operator *with scaling factor *s* = 1.0.

**Figure 6:**
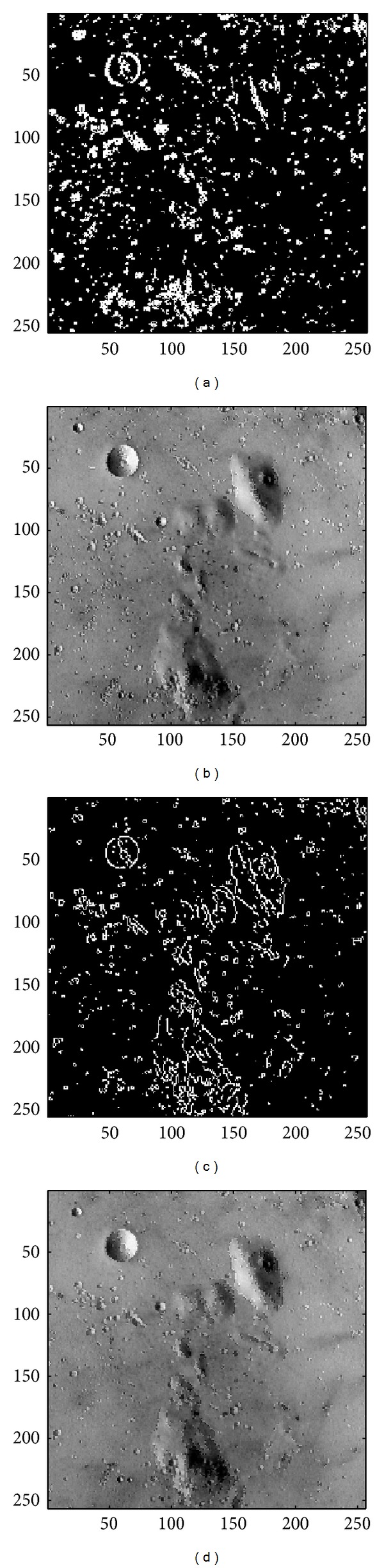
(a) Edges detected by HVD with *θ*_eth_ = 16 in the image “Moonsurface.” (b) Result by HVD with scaling factor *s* = 1.0. (c) Edges detected by* Canny operator *in the image “Moonsurface.” (d) Result by* Canny operator *with scaling factor *s* = 1.0.

**Figure 7:**
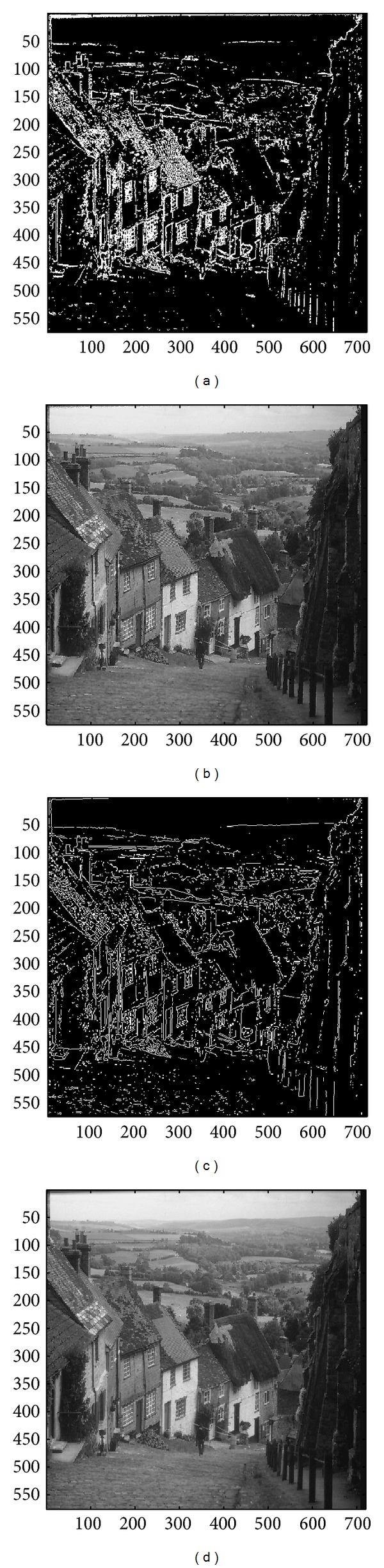
(a) Edges detected by HVD with *θ*_eth_ = 14 in the image “Goldhill.” (b) Result by HVD with scaling factor *s* = 1.0. (c) Edges detected by* Canny operator *in the image “Goldhill.” (d) Result by* Canny operator *with scaling factor *s* = 1.0.

**Figure 8:**
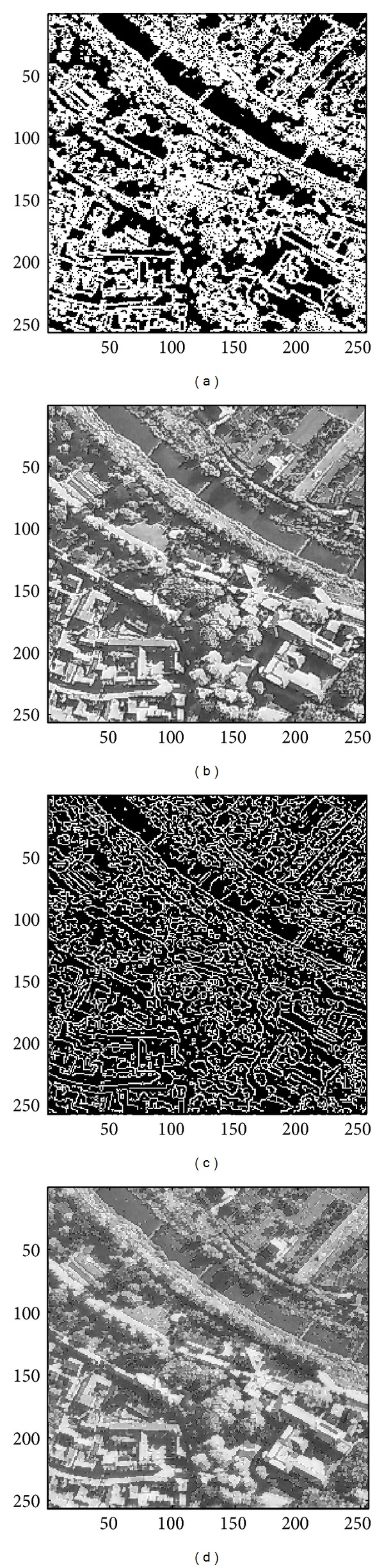
(a) Edges detected by HVD with *θ*_eth_ = 12 in the image “Aerial.” (b) Result by HVD with scaling factor *s* = 1.0. (c) Edges detected by* Canny operator *in the image “Aerial.” (d) Result by* Canny operator *with scaling factor *s* = 1.0.

**Figure 9:**
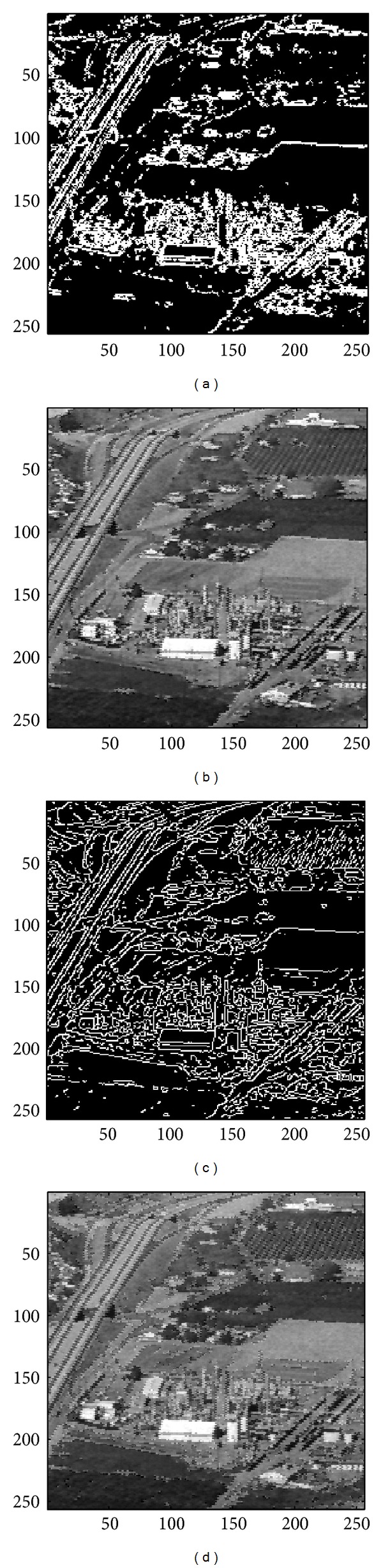
(a) Edges detected by HVD with *θ*_eth_ = 18 in the image “Chemicalplant.” (b) Result by HVD with scaling factor *s* = 1.0. (c) Edges detected by* Canny operator *in the image “Chemicalplant.” (d) Result by* Canny operator *with scaling factor *s* = 1.0.

**Figure 10:**
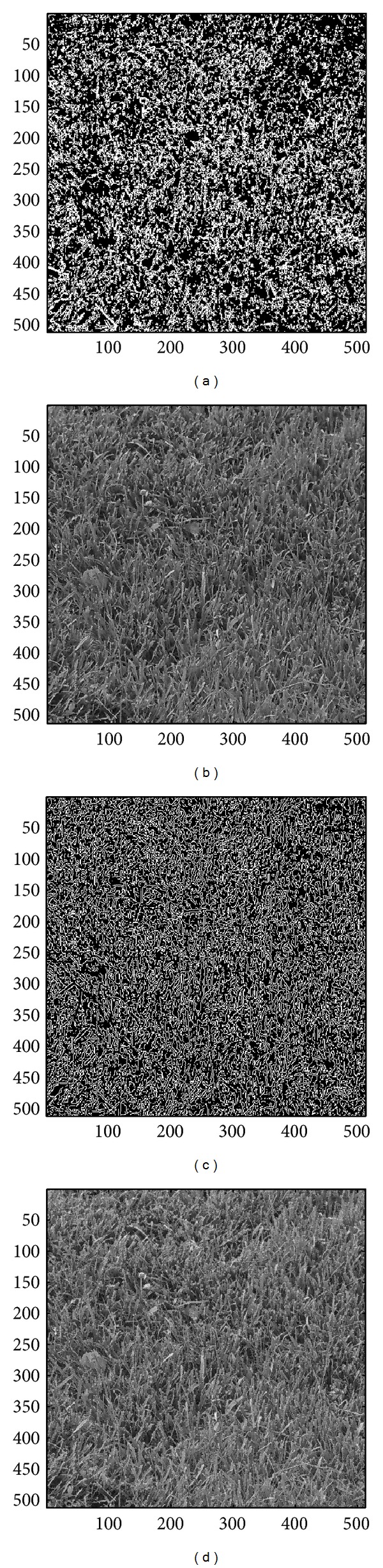
(a) Edges detected by HVD with *θ*_eth_ = 18 in the image “Grass.” (b) Result by HVD with scaling factor *s* = 1.0. (c) Edges detected by* Canny operator *in the image “Grass.” (d) Result by* Canny operator *with scaling factor *s* = 1.0.

**Figure 11:**
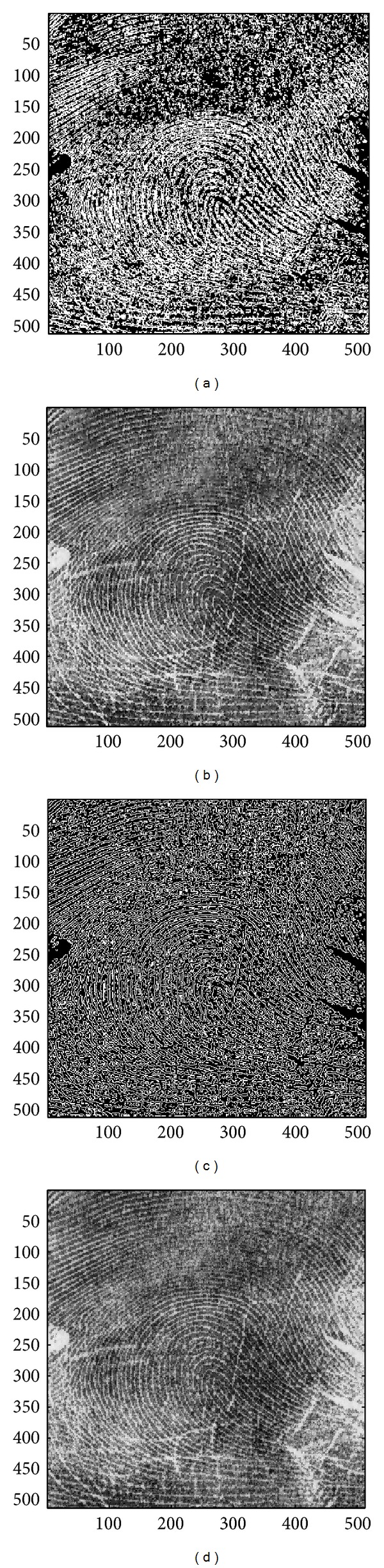
(a) Edges detected by HVD with *θ*_eth_ = 18 in the image “Finger.” (b) Result by HVD with scaling factor *s* = 1.0. (c) Edges detected by* Canny operator *in the image “Finger.” (d) Result by* Canny operator *with scaling factor *s* = 1.0.

**Figure 12:**
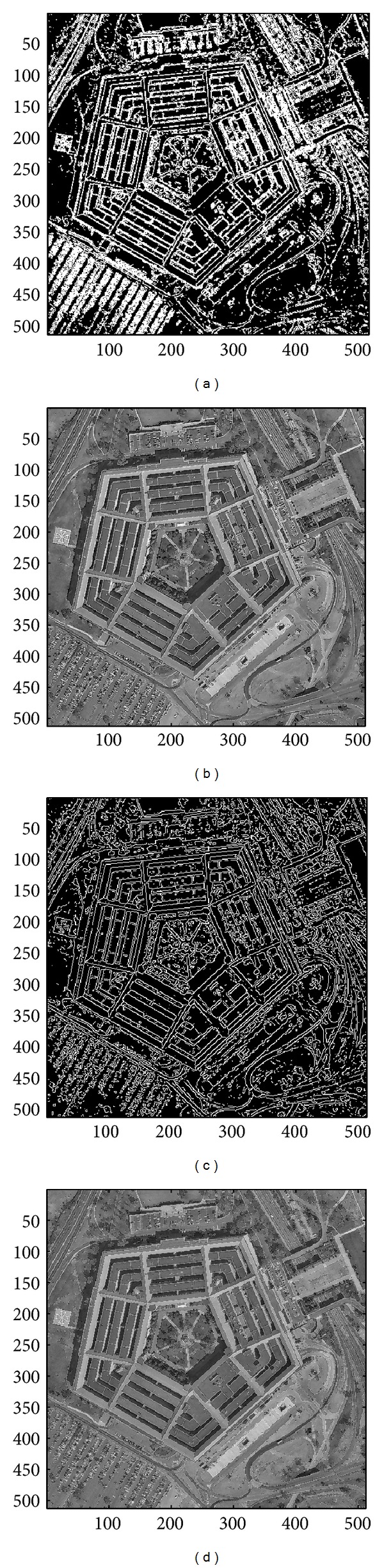
(a) Edges detected by HVD with *θ*_eth_ = 10 in the image “Pentagon.” (b) Result by HVD with scaling factor *s* = 1.0. (c) Edges detected by* Canny operator *in the image “Pentagon.” (d) Result by* Canny operator *with scaling factor *s* = 1.0.

**Figure 13:**
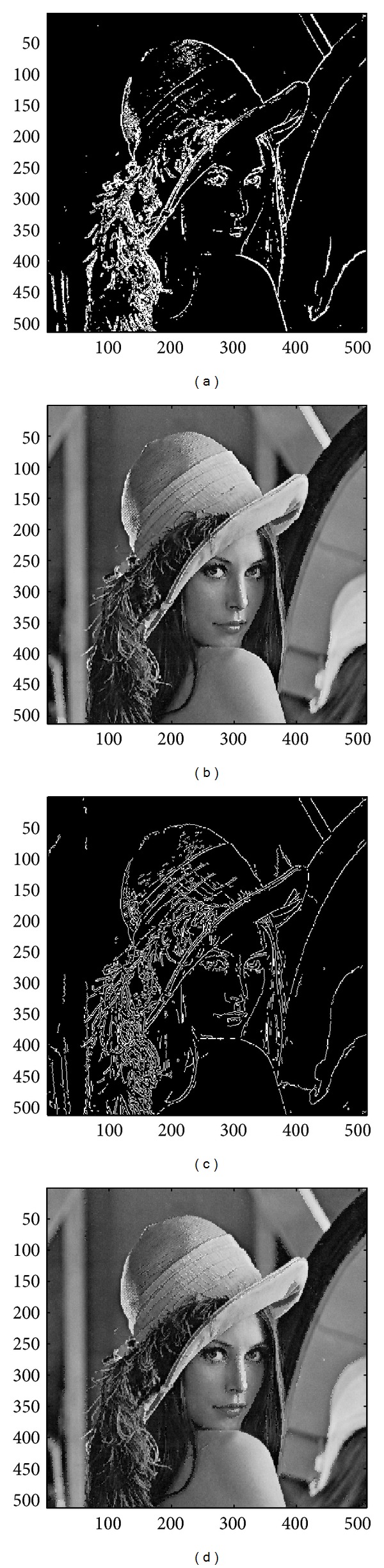
(a) Edges detected by HVD with *θ*_eth_ = 18 in the image “Lena.” (b) Result by HVD with scaling factor *s* = 1.0. (c) Edges detected by* Canny operator *in the image “Lena.” (d) Result by* Canny operator *with scaling factor *s* = 1.0.

In this subsection, the sharpened result by using the proposed approach for a set of nine test images in Figure 4, including three SAR images, one medical image and five natural images, will be given and evaluated in a subjective manner. We show in Figures 5 to 13 the results of the proposed approach. Among which, Figures 5(a) to 13(a) are those edge pixels picked out by using the* HVD* edge detector, while Figures 5(c) to 13(c) are obtained by that of* Canny operator*. Besides, Figures 5(b) to 13(b) are the sharpened image obtained by using the proposed approach with* HVD* edge detector, and Figures 5(d) to 13(d) are that of* Canny operator*. All the sharpened images in Figures 5 to 13 are obtained by setting the scaling factor *s* equal to 1.

As can be seen in Figures 5(b) and 5(d), the skeleton of the medical image “Neck” has become more conspicuous when compared with the original medical image in Figure 4(a). The same effect can be obtained by observing the contour of those cavities in Figures 6(b) and 6(d) for the test image “Moonsurface” in Figure 4(b). For the natural image “Goldhill,” we can observe the portion of the windows in Figures 7(b) and 7(d). The outline of the windows is more distinct than that of the original unsharpened image in Figure 4(c). In addition, we also show in Figures 8 and 9 the sharpened results obtained by using the proposed approach for the two remote sensed SAR test images “Aerial” and “Chemicalplant.” As can be seen in Figures 8(b) and 8(d), the contour of the house becomes more conspicuous after the sharpening process when compared with the original image in Figure 4(d). For the test image “Chemicalplant,” we see from Figures 9(b) and 9(d) that the contour of the factory and the tanks, as well as the road on the left side of the picture can all have a very good visual quality when compared with the original image in Figure 4(e). For the test image “Grass” in Figure 4(f), we can see in Figures 10(b) and 10(d) that a very distinct contour around the grass can be obtained after the sharpening process when compared with the original image. The results of the test image “Finger” are shown in Figures 11(b) and 11(d). As can be seen in Figures 11(b) and 11(d), the texture of the finger print has become quite obvious when compared with the original image in Figure 4(g).  Figure 12 shows the sharpened results of the test image “Pentagon,” an aerial image. A remarkable contour around the center of the buildings and the bridges, as well as the roads can be obtained after the proposed sharpening process (Figures 12(b) and 12(d)) when compared with that of the original image (Figure 4(h)). Finally, for the well known test image “Lena” (Figure 4(i)), we show in Figures 13(b) and 13(d) the sharpened results by using the proposed approach. As can be seen in Figures 13(b) and 13(d), the contour around her eyes and the contour of the hair have become very conspicuous after the sharpening process.

As can be seen in Figures 5 to 13, the contours of the sharpened images have become quite conspicuous when compared with that of the original image (Figure 4). Moreover, the result obtained by using the HVD edge detector (Figures 5(b) to 13(b)) also exhibits a better visual quality than that of using the Canny operator (Figures 5(d) to 13(d)). Obviously, a better visual perception on the texture or contour can be obtained during the image sharpening process if pixels on both sides of an edge can be adjusted simultaneously, that is, to increase the sharpness on both sides of an edge simultaneously. However, when we look into the edges detected by using the HVD edge detector (Figures 5(a) to 13(a)) and that of Canny operator (Figures 5(c) to 13(c)), we find that the width of an edge detected by using the Canny operator is subtle and would be only one-pixel in most of the cases, meaning that only pixels in one side of an edge will be adjusted. Therefore, the discontinuity can not be as distinct as that of using the HVD edge detector under the same scaling factor *s*.

### 4.2. Objective Performance Evaluation with PSNR

In addition to the subjective evaluation on the test images, most of the researches apply the peak signal to noise ratio (PSNR), an objective metric, as well to evaluate the objective quality of the proposed image sharpening algorithm. That is, we want to make the contour or outline of an image visually conspicuous, but with most of the content information kept unaltered. Therefore, we use the PSNR as a metric to check the difference of an image before and after the sharpening process. Moreover, we also investigate in experiments the PSNR of the proposed approach by using different scaling factors *s* with 0.5, 0.8, and 1.0, respectively. The results of the objective performance evaluation obtained by using the proposed approach are listed in Table 1. The maximal additive value Δ for individual test image is also listed in the last column of Table 1. As can be seen in Table 1, the sharpened images can still have a very good PSNR (all above 43.881 dB), which indicates that most of the information in the original image can be retained after the sharpening process. Therefore, a very good trade-off between the edge enhancement and the conservation of the original content can be reached by using the proposed approach.

### 4.3. Complexity of the Proposed Algorithm

The operation counts of the proposed approach are listed in Table 2. The first row of Table 2 indicates different kinds of operations used during the sharpening process in the proposed approach. For edge pixels detection process, the operation counts needed to perform the proposed horizontal and vertical difference (*HVD*) edge detection are listed in the second row of Table 2 [10]. It is noted that the operations in the second row are performed for each pixel in the image to be sharpened if the* HVD* is chosen for the edge detection. The operation counts of the low-pass filtering and edge sharpening process are given in the third row and the fourth row, respectively [10]. As the process of low-pass filtering and sharpening is applied only for pixels around edges or boundaries, it should be noted that these operation counts are required only for those edge pixels.

## 5. Conclusion

Aimed to find the additive magnitude automatically and adaptively, we propose a very simple yet effective three-step approach based on the Grey prediction theory for the sharpening of images in this paper. The proposed approach can adapt itself to the global and local statistics of the image to be sharpened. Extensive experiments on subjective and objective evaluations on PSNR show that the discontinuity can be made conspicuous with the proposed approach without losing much original information of the image to be sharpened, which demonstrates the superiority of the proposed approach.

## Figures and Tables

**Table 1:** Comparisons of the sharpened images with respect to original images in terms of PSNR.

Image	Dimension	Edge sharpening images												
		(a) HVD			(a) HVD with *θ*_Lpf_ ≥ 3			(b) Canny			(b) Canny with *θ*_Lpf_ ≥ 2			Δ
		*s* = 0.5	*s* = 0.8	*s* = 1.0	*s* = 0.5	*s* = 0.8	*s* = 1.0	*s* = 0.5	*s* = 0.8	*s* = 1.0	*s* = 0.5	*s* = 0.8	*s* = 1.0	
Neck	504 × 700	77.938	68.218	63.720	83.028	73.281	68.796	81.480	71.643	67.189	83.704	73.878	69.420	38
Moon surface	256 × 256	70.265	60.698	56.194	75.289	65.728	61.234	75.706	66.163	61.674	79.315	69.764	65.280	41
Gold hill	720 × 576	73.264	63.575	59.054	76.332	66.632	62.104	77.978	68.261	63.742	88.846	79.145	74.616	34
Aerial	256 × 256	57.342	47.837	47.837	57.781	48.276	43.881	62.617	53.076	48.629	63.789	54.240	49.781	43
Chemical plant	256 × 256	67.366	57.742	53.247	69.263	59.638	55.147	68.819	59.155	54.653	70.337	60.679	56.174	38
Grass	512 × 512	67.633	57.979	53.434	69.682	60.028	55.482	69.336	59.706	55.168	70.562	60.930	56.392	31
Finger	512 × 512	60.275	50.685	46.162	61.302	51.712	47.189	63.510	53.933	49.414	64.274	54.697	50.177	39
Pentagon	512 × 512	66.284	56.653	52.128	67.386	57.755	53.231	71.255	61.647	57.142	72.705	63.093	58.586	33
Lena	512 × 512	76.111	66.575	62.161	78.282	68.720	64.268	78.625	69.088	64.676	79.357	69.812	65.393	39

“*s*” is a scaling factor between 0 and 1.

**Table 2:** Operation counts of the proposed approach.

Operations	APU	MPU/DIV	ABS	COMP
HVD	≤2	0	≤2	≤2
Low pass filtering	7	0	0	1
Edge sharpening	9	4	0	1

HVD is required for each pixel.

LPF and Edge sharpening is for edge pixel only.
